# The Dynamic ATP-Driven Mechanism of Bacterial Protein Translocation and the Critical Role of Phospholipids

**DOI:** 10.3389/fmicb.2019.01217

**Published:** 2019-06-19

**Authors:** Ian Collinson

**Affiliations:** School of Biochemistry, University of Bristol, Bristol, United Kingdom

**Keywords:** SecA ATPase, bacteria, SecY translocon, lipids, protein secretion

## Abstract

Protein secretion from the cell cytoplasm to the outside is essential for life. Bacteria do so for a range of membrane associated and extracellular activities, including envelope biogenesis, surface adherence, pathogenicity, and degradation of noxious chemicals such as antibiotics. The major route for this process is *via* the ubiquitous Sec system, residing in the plasma membrane. Translocation across (secretion) or into (insertion) the membrane is driven through the translocon by the action of associated energy-transducing factors or translating ribosomes. This review seeks to summarize the recent advances in the dynamic mechanisms of protein transport and the critical role played by lipids in this process. The article will include an exploration of how lipids are actively involved in protein translocation and the consequences of these interactions for energy transduction from ATP hydrolysis and the trans-membrane proton-motive-force (PMF).

## Protein Secretion Through the Bacterial Sec Machinery

Protein secretion, from the cell cytoplasm to the outside, is essential for life. Bacteria secrete proteins for enveloping biogenesis, surface adherence, and pathogenicity and degrading noxious chemicals (including antibiotics), among a range of many other membrane and extracellular activities. The major route for protein secretion is *via* the ubiquitous Sec translocon: a conserved hetero-trimeric core-complex of the inner membrane. This machinery is also responsible for membrane protein insertion, whereby proteins containing **T**rans-**M**embrane α-**H**elices (TMH) are threaded laterally into the bilayer rather than across it. Therefore, the interaction of the machinery with lipids is critical in that they interface with the protein complex through which proteins cross and enter the membrane. Moreover, it turns out that phospholipids play direct and critical roles in the active energy transducing process driving protein transport.

**Figure 1:**
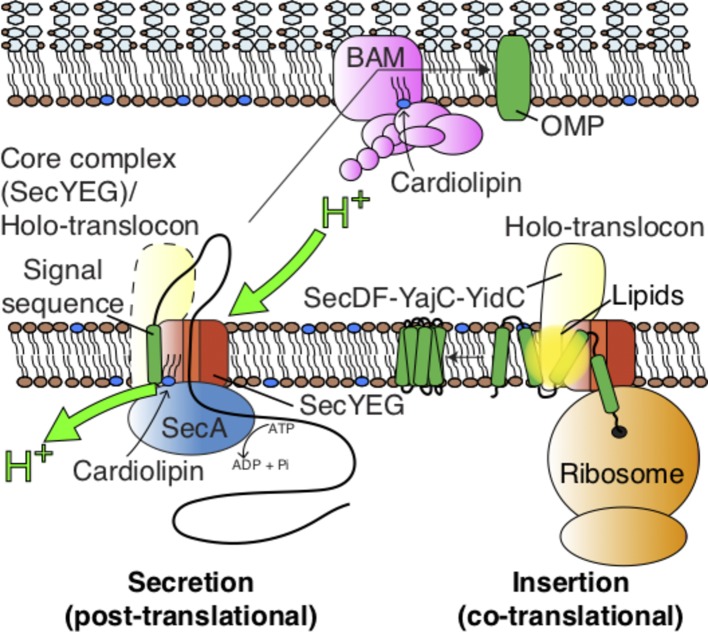
Pathways for protein transport across and into the Gram-negative inner membrane and further across the periplasm for outer-membrane insertion and folding. The figure also highlights the action of lipids on the SecYEG-SecA complex (see Figure 3), the holo-translocon (HTL), and the BAM complex.

Secretory and membrane proteins are targeted to the Sec machinery with the aid of an N-terminal signal sequence (Chang et al., 1978; Walter et al., 1981b). These proteins then translocate through the apparatus in an unfolded conformation (Arkowitz et al., 1993), either during their synthesis (co-translationally), or afterwards (post-translationally). The former is a ubiquitous process in which the signal sequence at the N-terminus of the nascent polypeptide emerging from the ribosome exit tunnel is recognized by the signal recognition particle (SRP) and targeted to the membrane-associated SRP-receptor (Walter et al., 1981a,b,c; Poritz et al., 1990). The ribosome nascent chain complex (RNC) is then shuttled to the Sec complex for translocation (Jomaa et al., 2016), where the growing polypeptide is forced through the membrane. In the bacterial model *Escherichia coli* (Figure 1), the co-translational pathway is generally utilized for membrane protein insertion (Ulbrandt et al., 1997; Müller et al., 2001), while transport across the membrane (secretion) tends to be post-translational. In the latter process, cytosolic chaperones, such as SecB, deliver the pre-protein in a translocation competent (unfolded) state to the SecA motor protein and channel complex SecYEG for secretion (Hartl et al., 1990; Cranford-Smith and Huber, 2018).

## Structure of the Core-Translocon

The pathway for translocation of the mature region of the pre-protein – the protein-conducting channel – is formed through the center of SecY, between two pseudo-symmetrical halves, each with five TMHs (Van den Berg et al., 2004; Cannon et al., 2005). When at rest, the channel is kept sealed by a short α-helix (the plug) and a sphincter of six hydrophobic residues, usually isoleucine (Van den Berg et al., 2004). Separation of the two halves opens a channel across the membrane (for secretion) as well as a lateral gate (LG) for entry of TMHs into the bilayer (insertion). Multiple rounds of ATP hydrolysis and the trans-membrane proton-motive-force (PMF) then drive protein transport across the membrane (Brundage et al., 1990; Schiebel et al., 1991; Economou and Wickner, 1994).

## Mechanism of Seca-Driven Protein Translocation

Today, we understand a great deal about the structure and activity of Sec machinery, particularly the bacterial counterpart (almost exclusively through the study of the *E. coli* system). During SecA-driven secretion, the association of SecA with the pre-protein causes the signal sequence to bind at the LG of SecY, at the interface with the lipid bilayer (Hizlan et al., 2012; Li et al., 2016). Many studies have shown that this interaction causes a conformational change in both SecA and the protein channel (Hizlan et al., 2012; Corey et al., 2016; Li et al., 2016), and a priming of SecA for increased ATPase activity (Gouridis et al., 2009; Robson et al., 2009; Gold et al., 2013). In this “unlocked” state, the channel opens slightly and the plug, which helps keep the Sec-complex closed, retracts from its central position (Zimmer et al., 2008; Hizlan et al., 2012; Corey et al., 2016; Li et al., 2016).

**Figure 2:**
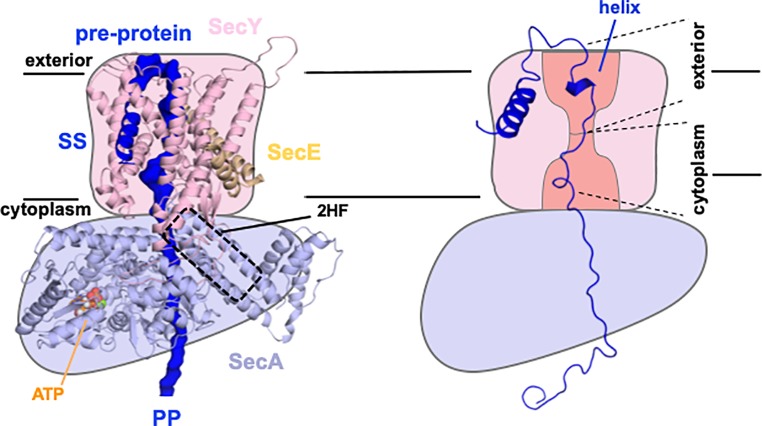
Sec control of protein folding as a driver for transport. Model structure of SecYEG-SecA^ATP^ based on Li et al. (2016) with key features labeled accordingly; SS, signal sequence; 2HF, 2 helix finger (left). Schematic of same (right), showing only the simulated structure of the pre-protein, with a partial folding in the exterior cavity (helix) and unfolded regions in the cytoplasmic cavity and hence favoring transport to the exterior, as required. Adapted from figures shown previously, used with permission (Corey et al., 2019).

Our understanding of this reaction has been aided by recent single-molecule fluorescence studies (Allen et al., 2016; Fessl et al., 2018). These applications enable the dissection and analysis of different stages of the reaction, which would otherwise be blurred in the reaction ensemble. The analysis demonstrates that the initiation process requires the signal sequence and mature regions of the pre-protein, as well as ATP (Fessl et al., 2018). The initiation involves an ATP-driven transport step, independent of pre-protein length, likely to be the intercalation of the signal sequence and early mature regions of the polypeptide (presumably as a loop with the N-terminus pointing towards the cytosol; Figures 1, 2) into the “unlocked” translocon, as was previously proposed (Hizlan et al., 2012). At this point, the ATPase becomes fully activated and translocation across the membrane can begin through SecYEG; the kinetics of which depend on the length of the substrate (see Fessl et al., 2018 and Figure 6 therein; Tomkiewicz et al., 2006).

## Model For Protein Translocation

For the transport process *per* se, a stochastic Brownian ratchet model for the ATP-driven reaction has been proposed wherein the free energy available from ATP binding and hydrolysis helps bias the random diffusional flow of polypeptide to favor an outward direction (see Figure 8 and associated movie in Allen et al., 2016).

A follow-up study proposed how this stochastic process could be further enhanced. ATP-dependent control of protein folding has been well documented in the protein chaperone field (Clarke, 1996). We have introduced this concept into the protein transport field whereby the translocon utilizes the hydrolytic cycle of ATP to exert an asymmetric control of pre-protein folding (Corey et al., 2019). Preventing partial folding at the cytosolic interface of the ATPase SecA with SecY, while enabling it at the outward facing exit site, would prevent back sliding of the translocating polypeptide (Figure 2), thus favoring outward flow of the pre-protein. This concept is consistent with independent findings that the folding propensity (or lack of it) of the mature regions of the pre-protein has profound effects on the secretion efficiency (Gonsberg et al., 2017; Jung and Tatzelt, 2018; Tsirigotaki et al., 2018).

An alternative “push-and-slide” model invokes both diffusional and ATP-driven power-stroke components, involving the 2-helix finger (2HF) motif of SecA moving up and down to physically push polypeptides across the membrane (Erlandson et al., 2008; Zimmer et al., 2008; Bauer et al., 2014). A recent follow-up study, based on single-molecule fluorescence, confirms that the 2HF is indeed conformationally mobile throughout the ATP-driven transport cycle (Catipovic et al., 2019). The differences in fluorescence were equated to a very large apparent change in energy transfer efficiency (~0.1 to ~0.9). The main contention here is if the observations are really due to **F**örster **R**esonance **E**nergy **T**ransfer (FRET), or to **P**rotein-**I**nduced **F**luorescent **E**nhancement (PIFE; Stennett et al., 2015). In the former case, this would require an extraordinarily large movement of >20 Å. Otherwise, the affect may be due to changes of the dye environment, for example, the formation of steric constraints at alternative conformations. This phenomenon is a known feature of some fluorescent reporters, particularly Cy3 (Stennett et al., 2015), used in this new study (Catipovic et al., 2019). Consequently, more subtle movements could also be responsible for these large fluorescent fluctuations, which would be more concordant with the limited space available for the 2HF to move (Zimmer et al., 2008; Li et al., 2016).

Either way, whether a diffusional ratchet (Allen et al., 2016) or power stroke/diffusional hybrid (Bauer et al., 2014; Catipovic et al., 2019) is at play, the core ATP-driven process is further stimulated by the PMF (Brundage et al., 1990) in order to achieve the high rates of secretion required for rapid growth. Presently, it is not known if or how the electrical (Δψ) or chemical (ΔpH) components of the PMF achieve this enhancement. The PMF may indeed also operate to favor the outward flow of polypeptide in a Brownian ratchet-type mechanism.

During the final stages of transport, the signal sequence of the pre-protein is proteolytically cleaved to release the mature protein on the other side of the membrane (Josefsson and Randall, 1981). The terminal closure of the translocon is apparently independent of ATP (Fessl et al., 2018).

## The Holo-Translocon (HTL)

To complicate matters further, the SecYEG core complex also associates with a number of accessory proteins: the membrane protein “insertase” YidC and the sub-complex SecDF (Duong and Wickner, 1997; Schulze et al., 2014). In a large number of cases, including *E. coli*, this complex also contains the YajC protein of obscure function (Duong and Wickner, 1997; Schulze et al., 2014). YidC facilitates the lateral insertion of TMHs from SecY into the bilayer (Houben et al., 2000; Samuelson et al., 2000; Scotti et al., 2000; Kumazaki et al., 2014), while SecDF makes an additional use of the PMF to help drive the transport of secretory proteins (Arkowitz and Wickner, 1994; Duong and Wickner, 1997; Tsukazaki et al., 2011; Botte et al., 2016; Furukawa et al., 2017). Thus, the resultant super-complex – the **H**olo-**T**rans**L**ocon (HTL) – associates with co-translating ribosomes for efficient membrane protein insertion and SecA for ATP/PMF-driven secretion (Figure 1; Schulze et al., 2014; Komar et al., 2016).

## Transport Through the Cell Envelope

For many proteins, transport across the inner membrane is only the first step. Following passage through the Sec-translocon proteins are either retained in the cell envelope or find their way to the external medium. Gram-negative bacteria have the added complexity of an outer membrane with an inter-membrane periplasm containing the peptidoglycan layer. Therefore, proteins must either be folded and retained in the periplasm or be further trafficked into or across the outer membrane. This is no mean feat.

There are a number of periplasmic shock proteins that are transported to the periplasm and folded in enormous quantities, e.g., Spy and HdeI (Tapley et al., 2009; Quan et al., 2011). Moreover, the demand for the insertion and folding of β-barrel **O**uter **M**embrane **P**roteins (OMPs) in rapidly dividing cells is vast. The process is facilitated by the periplasmic chaperones SurA and Skp (Sklar et al., 2007; McMorran et al., 2013), which presumably collect proteins as they emerge from the translocon for folding or for delivery to the outer membrane. How the chaperoned OMPs negotiate the peptidoglycan layer is unclear. We know that when they get there, they are welcomed by the β-**B**arrel **A**ssembly **M**achinery (BAM) – a complex of five proteins BamABCDE, responsible for the insertion and folding of OMPs (Figure 1; Voulhoux et al., 2003; Wu et al., 2005). But how this is achieved for very large fluxes of proteins, without aggregation, and in the absence of an energy source is not easily reconciled. Many structures of the BAM complex have been determined (Bakelar et al., 2016; Gu et al., 2016; Iadanza et al., 2016); despite this, the mechanism for energy-independent OMP insertion and folding has yet to emerge.

## The Role of Specific Phospholipids in Protein Transport

While the dynamic mechanism for protein secretion through the protein machinery of the bacterial energy conserving, inner membrane has been the focus of our attention, it is becoming increasingly clear that the resident lipids also play a critical role in the transport proteins across, as well as into the membrane (Figure 1).

This post-translational reaction has been known for many years to require acidic phospholipids. Mutants defective in acidic phospholipid – cardiolipin (CL) and phosphatidyl-glycerol (PG) – synthesis have protein export deficiencies (Tommassen et al., 1989). Moreover, these lipids are required for functional association of SecA to the inner membrane (Hendrick and Wickner, 1991). Later work showed that the CL and, to a lesser extent, PG are important for stability of the SecYEG complex and to stimulate SecA ATPase activity (Bessonneau et al., 2002; Gold et al., 2010) – but the mechanism of action was unclear. Recent progress on this subject is beginning to unravel the mysterious action of this unique lipid.

**Figure 3:**
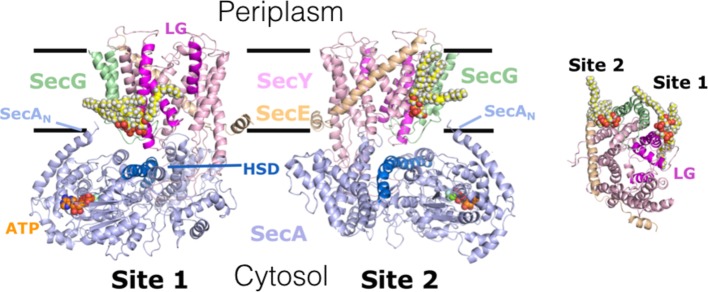
Interaction of cardiolipin (CL) with the bacterial translocon. 2 CL (yellow, white, and red spheres) – binding sites 1 & 2 – on SecYEG; identified by CGMD, validated and shown to be critical for ATP and PMF-driven secretion (Corey et al., 2018). Left and middle, side view of SecYEG bound to the motor ATPase SecA; right, cytosolic view of SecYEG alone. In SecA: ATP, SecA_N_ – N-terminus; HSD (including the 2HF) – helical scaffold domain, at the entrance to the protein channel in SecY. In SecYEG: LG – lateral gate, which opens during transport of proteins across and into the membrane. Adapted from Corey et al. (2018), used with permission.

Course-Grain Molecular Dynamics (CGMD) simulations have identified SecYEG sites, which transiently interacted with CL, which were validated empirically (Figure 3; Corey et al., 2018): native mass spectrometry demonstrated that variants in which the positive surface charges of the putative binding sites were diminished, bound CL less effectively. Remarkably, it turns out that these specific CL interactions confer the stimulation of SecA ATPase activity and PMF enhancement of secretion (Figures 1, 3; Corey et al., 2018); the latter may be achieved by proton carriage by the lipid itself. If true, this would be the first description of a direct involvement of a phospholipid in the process of energy coupling.

Furthermore, the interaction of the translocon with CL has profound consequences for the structure of the protein complex. Interestingly, specific sites on SecYEG, used to monitor the opening and closure of the protein channel (Fessl et al., 2018), are strongly dependent on CL for SecA promoted channel opening (Figure 5 and Supplementary Figure 2 in Corey et al., 2019).

## A Lipid Pool in the HTL

Co-translational transport of proteins into the membrane occurs through the HTL (see above; Figure 1). Lipids, again CL in particular, are required to stabilize the holo-complex (Schulze et al., 2014), and critically, lipids also form an encapsulated pool at its center (Figure 1; Botte et al., 2016; Martin et al., 2019). This remarkable feature could provide an enclosed lipidic environment to promote efficient membrane protein insertion and assembly, protecting the translocating membrane protein from aggregation and proteolysis. This is a familiar concept for promoting folding of globular proteins within a chamber of the chaperonin GroEL (Ranson et al., 1997; Xu et al., 1997).

## Other Protein Translocation Systems

It is very interesting that other protein translocation systems have also been implicated in the association with CL, including the BAM complex of the outer membrane (Figure 1; Chorev et al., 2018), and the mitochondrial Tim23 import machinery (Malhotra et al., 2017), but apparently not the TAT machinery responsible for the export of fully folded proteins in bacteria (Rathmann et al., 2017). Given the well-known dependence of CL for many proton translocating energy transducing systems, such as the ATP synthase (Duncan et al., 2016), and the electron transfer chain complexes I, III, and IV (Fry and Green, 1981; Pfeiffer et al., 2003; Fiedorczuk et al., 2016; Malkamäki and Sharma, 2019), one could surmise a critical role for proton-driven protein transport too.

## Conclusion

Finally, our understanding of the dynamic mechanism underlying ATP-driven secretion through the Sec machinery is approaching clarity, while its augmentation by the PMF is a mystery shortly to be resolved, after nearly 30 years since its discovery (Brundage et al., 1990; Schiebel et al., 1991). In this context, CL seems to play essential and multifarious roles, for the structure and for both ATP and PMF-driven protein translocation activity. This warrants further investigation and exploitation. The essential lipid-protein interface could be a prime target for infiltration by small molecules for prospective antibiotic development.

## Author Contributions

The author confirms being the sole contributor of this work and has approved it for publication.

### Conflict of Interest Statement

The author declares that the research was conducted in the absence of any commercial or financial relationships that could be construed as a potential conflict of interest.
